# Bibliography

**Published:** 1902-03

**Authors:** 


### Bibliography.

Principles and Practice of Operative Dentistry. By John Sayre Marshall, M.D. (Syr. Univ.), Dental Surgeon United States Army, President Army Examining Board for Dental Surgeons. J. B. Lippincott Company, Philadelphia and London, 1901.

This work, on the very important branch of operative dentistry, is presented to practitioners of dental surgery by the author, and, to use his own language, "it has been his endeavor so to present the subject-matter as to give the student a comprehensive view of the principles and practice of operative dentistry, arranged in a natural and orderly sequence. . . . The methods of constructing artificial crowns and bridge-work have not been included in this volume, because in the opinion of the author they properly belong to the department of prosthetic dentistry. The subject of orthodontia has also been excluded for the reason that this branch has assumed the proportions of a separate specialty."

There are forty-two chapters in the book, beginning with the "Classification and Descriptive Anatomy of the Teeth" and closing with "Extraction of Teeth." Between these all the important subjects connected with what is known as operative dentistry are considered in detail.

The illustrations are profuse, consisting of seven plates and seven hundred and twenty-five illustrations. Many of these are exceptionally good, especially those photomicrographs, prepared by Dr. Vida A. Latham, of "normal and pathological dental tissues and of bacteriologic specimens." Others by Drs. Andrews, Miller, Williams, Vincentini, of Naples, Noyes, Cryer, and Mr. James S. Shearer make up a collection rarely found in any single book.

Excluding plastics and inlays, there are sixty-one pages devoted to preparation of cavities and filling teeth, or about one-tenth of the reading matter of the volume. This fact, if it have any importance, shows the gradual loss which this principal operation has undergone in the past fifty years,—not that it is undervalued at the present time, but that so much of the collateral sciences have been added that it has taken, in the estimation of many, a subordinate place in the curriculum. This the writer must regard as an unfortunate conclusion. This is, however, the legitimate result of the advocacy of the medical degree in the practice of dentistry. The skilled men in the latter will eventually be superseded by the imperfectly trained.

The publishers have spared nothing to make this volume worthy the subject, and the author is to be commended for his effort to make a compilation covering the important points connected with the daily practice over the dental chair.

Questions and Answers. Embracing the Curriculum of the Dental Student, divided into Three Parts. By Ferdinand J. S. Gorgas, A.M., M.D., D.D.S., Author of "Dental Medicine," etc., Professor of the Principles of Dental Science, Oral Surgery, etc., in the University of Maryland, Dental Department, Baltimore. Published by P. Blakiston's Son & Co., 1012 Walnut St., Philadelphia.

Whenever there is a want to be supplied some one steps in to meet the demand. The author doubtless recognized that the period was ripe for a carefully arranged book of questions and answers, and prepared the first edition, published in three thin volumes. This edition, numbering four thousand five hundred, has been practically exhausted, necessitating a thorough revision and enlargement. This the author has now completed in a large volume of five hundred and forty pages.

It is impossible to examine this book and not feel impressed with the industry of the author. To prepare a series of questions upon any given subject where the compiler is very familiar with the topic treated is a serious task, but when this is complicated with many subjects comprising the entire curricula of the schools, it becomes formidable. That the author has been measurably successful must be conceded, and the questions and answers are, upon the whole, satisfactory.

The first edition was planned upon the supposition that the various studies extended over three years, and he therefore divided his book into the supposed work of the Freshman, Junior, and Senior Years. This is carried out, in extended form, in this, the second edition. The method has some advantages, but as, probably, no dental school carries all the subjects of the curriculum through three years, the plan will not prove satisfactory, and it would seem better to abandon this arrangement in future editions.

In such a laborious task as this book represents it could not be expected that its teaching would harmonize with that of all the dental colleges, and, therefore, such a book must necessarily fail to be universally acceptable. A critical examination as to the truth or error contained would be difficult and perhaps impossible, but the reviewer, in glancing through its pages has met with a few statements that will lead to erroneous teaching. The following are typical: It is somewhat surprising that a teacher of materia medica would recommend equal parts of aconite and iodine for the treatment of pericementitis. A close study of the properties of aconite and its relation to the function of the sensory nerves in counterirritation should have caused the author to hesitate in recommending this powerful paralyzing agent.

Again, in the use of chloride of zinc for obtunding sensitive dentine. There may be room for a difference of opinion here, but it is an established fact that the vitality of the pulp is endangered by its use.

The author's treatment of pulpless teeth by pyrozone will hardly be satisfactory to those who doubt the propriety of using an active irritant in canals with dead pulps.

The author's idea of abrasion is certainly erroneous. He regards the wasting as peculiarly the result of mechanical effect. The fact that acids are a prominent factor is not regarded.

If the author anticipated that this book would be used as a text-book in dental colleges, it is feared he will be disappointed. That it has been used by a certain class is very evident from the large sale of the first edition. It is inconceivable that any teacher would adopt this in his class work.

Quiz teachers will find the book a present help, and to boards of examiners it will solve many difficult problems. The student, however, who depends upon question and answer for his knowledge of any subject will suffer shipwreck eventually, for no study can be made part of the mental treasures by any such superficial pabulum.

If this volume be put to its legitimate use it will become of very great value, and for this it can be cordially recommended. The question, What is legitimate and what not? must be left to the individual, but in the reviewer's opinion the proper place is not in the hands of the student; neither should it be needed by a member of the State Board, for when the latter requires this help he at once acknowledges his ignorance of the subject.

The work of the publisher is satisfactory in every respect.

